# 315. An Interpretable Machine Learning Model Using The Gut Microbiome to Predict Clinical *E. faecium* Infection in Human Stem-Cell Transplant Recipients

**DOI:** 10.1093/ofid/ofad500.386

**Published:** 2023-11-27

**Authors:** Ramtin Zargari Marandi, Jens Christian Nørgaard, Emma Elizabeth Ilett, Marc Noguera Julian, Roger Paredes, Jens D Lundgren, Mette Jørgensen, Henrik Sengeløv

**Affiliations:** Rigshospitalet, Copenhagen University Hospital, Copenhagen Ø, Hovedstaden, Denmark; Centre of Excellence for Health, Immunity and Infections (CHIP), Rigshospitalet, Copenhagen University Hospital, Copenhagen, Denmark, Copenhagen, Hovedstaden, Denmark; Centre of Excellence for Health, Immunity and Infections (CHIP), Rigshospitalet, Copenhagen University Hospital, Copenhagen, Denmark; Center for Basic Metabolic Research (CBMR), Copenhagen University, Denmark, Copenhagen, Hovedstaden, Denmark; IrsiCaixa Institute for AIDS Research, Badalona, 08916 Catalonia, Spain, Barcelona, Catalonia, Spain; Hospital Universitari Germans Trias i Pujol, Badalona, Catalonia, Spain, Copenhagen, Hovedstaden, Denmark; Centre of Excellence for Health, Immunity and Infections (CHIP), Rigshospitalet, Copenhagen University Hospital, Copenhagen, Denmark; Department of Clinical Medicine, Copenhagen University, Copenhagen, Denmark, Copenhagen, Hovedstaden, Denmark; Centre of Excellence for Health, Immunity and Infections (CHIP), Rigshospitalet, Copenhagen University Hospital, Copenhagen, Denmark, Copenhagen, Hovedstaden, Denmark; Copenhagen University Hospital, Rigshospitalet, Copenhagen, Denmark, Copenhagen, Hovedstaden, Denmark

## Abstract

**Background:**

Enterococcus faecium (E. faecium) is a commensal bacterium found in the gastrointestinal tract. In immunocompromised patients, such as hematopoietic stem cell transplant (HSCT) recipients, it is a frequent course of extraintestinal infections. Identifying HSCT patients who are predisposed to developing such infections could enable the development of targeted strategies to reduce their risk. We aimed to develop a machine learning (ML) model to predict E. faecium infection in HSCT patients based on gut metagenomic data.

**Methods:**

In a cohort of HSCT recipients, fecal samples were collected around the time of transplantation. The samples generated metagenomic data derived from high-throughput DNA sequencing. The patients were followed for clinical infections up to 30 days post-sampling. As an interpretable ML model, we utilized the Q-Lattice, implementing symbolic regression to identify interactions among variables. We chose 20 bacterial species and 37 antibiotic resistance genes (ARGs) within the gut, associated with clinical *E. faecium* infection. We used 80% of the data to train the model to predict clinical *E. faecium* infection. The model was evaluated on the remaining 20% of the data (validation set) using the area under the curve (AUC) and confusion matrix.

**Results:**

Twenty-nine clinical *E. faecium* infections were observed within 30 days after the sampling of 656 gut material retrieved from 276 HSCT recipients. We found three bacterial species (*Bacteroides dorei*, *Blautia wexlerae*, *Fusicatenibacter saccharivorans)* and one ARG (*patA)* that contributed to predicting the *E. faecium* infection (Fig 1). All features in this final model, except for *patA*, were negatively associated with *E. faecium* infection (Spearman’s |rho| < 0.2). The ML model demonstrated 100% sensitivity and negative predictive value in predicting the infection in both the development and validation sets (AUC of 0.77 and 0.76, respectively).

an overview of the prediction model

**Figure:**
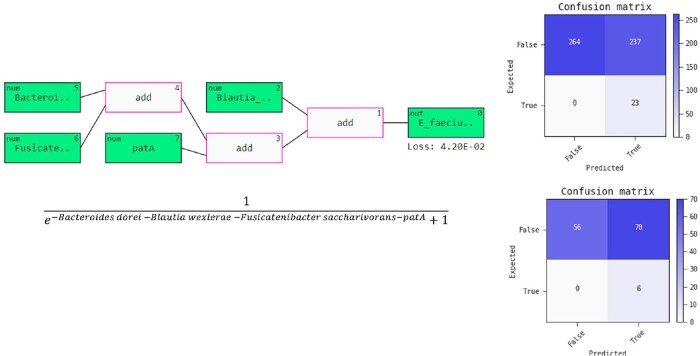

Prediction model for E. faecium Infection as a block diagram (on top) where the predictors Bacteroides dorei, Blautia wexlerae, Fusicatenibacter saccharivorans, and patA in the gut were selected by the model and the model representation as a closed-form equation (bottom). The confusion matrices display model performance on the development set (top) and validation set (bottom) where true denotes positive (infected) and false denotes negative (not infected). Also “expected” here means “truth” or “actual” class. Software package, Feyn in Python, was used for the model development and visualizations.

**Conclusion:**

The ML approach identified complex relationships between multiple microbial agents in predicting the risk of the infection. The predictive results were internally validated but requires external validation. These findings suggest that ML analyses of the gut microbiome is useful in predicting clinical *E. Faecium* infection.

**Disclosures:**

**Roger Paredes, M.D, Ph.D.**, Atea: Advisor/Consultant|Gilead: Advisor/Consultant|Lilly: Advisor/Consultant|MSD: Advisor/Consultant|MSD: Grant/Research Support|Pfizer: Advisor/Consultant|Roche: Advisor/Consultant|Theratechnologies: Advisor/Consultant|ViiV: Advisor/Consultant|ViiV: Grant/Research Support

